# Immediate impact of the COVID-19 pandemic on CTSA TL1 and KL2 training and career development

**DOI:** 10.1017/cts.2020.504

**Published:** 2020-06-29

**Authors:** Wayne T. McCormack, Miriam A. Bredella, David H. Ingbar, Rebecca D. Jackson, Emma A. Meagher, Cynthia D. Morris, Joan D. Nagel, Susan Pusek, Doris M. Rubio, Kathryn Sandberg, H. William Schnaper, Joel Tsevat, Jason G. Umans, Scott McIntosh

**Affiliations:** 1 Clinical & Translational Science Institute, Department of Pathology, Immunology and Laboratory Medicine, College of Medicine, University of Florida, Gainesville, FL, USA; 2 Harvard Catalyst, The Harvard Clinical and Translational Science Center, Harvard Medical School, Boston, MA, USA; 3 Clinical and Translational Science Institute Research Education, Career Development, and Training Core, Department of Medicine, University of Minnesota School of Medicine, Minneapolis, MN, USA; 4 The Center for Clinical and Translational Science, Department of Internal Medicine, The Ohio State University, Columbus, OH, USA; 5 Institute for Translational Medicine and Therapeutics, Perelman School of Medicine, University of Pennsylvania, Philadelphia, PA, USA; 6 Oregon Clinical and Translational Research Institute, Oregon Health & Science University, Portland, OR, USA; 7 National Center for Advancing Translational Sciences, National Institutes of Health, Bethesda, MD, USA; 8 NC TraCS Institute, University of North Carolina at Chapel Hill, Chapel Hill, NC, USA; 9 Institute for Clinical Research Education, Clinical and Translational Science Institute, University of Pittsburgh, Pittsburgh, PA, USA; 10 Georgetown-Howard Universities Center for Clinical and Translational Science, Washington, DC, USA; 11 Northwestern University Clinical and Translational Sciences (NUCATS) Institute, Northwestern University, Chicago, IL, USA; 12 Institute for the Integration of Medicine and Science, Center for Research to Advance Community Health, Department of Medicine, Long School of Medicine, University of Texas Health Science Center at San Antonio, San Antonio, TX, USA; 13 Center for Leading Innovation and Collaboration, Department of Public Health Sciences, University of Rochester Medical Center, Rochester, NY, USA

**Keywords:** TL1, KL2, COVID-19, research training, career development

## Abstract

Clinical and Translational Science Award (CTSA) TL1 trainees and KL2 scholars were surveyed to determine the immediate impact of the COVID-19 pandemic on training and career development. The most negative impact was lack of access to research facilities, clinics, and human subjects, plus for KL2 scholars lack of access to team members and need for homeschooling. TL1 trainees reported having more time to think and write. Common strategies to maintain research productivity involved time management, virtual connections with colleagues, and shifting to research activities not requiring laboratory/clinic settings. Strategies for mitigating the impact of the COVID-19 pandemic on training and career development are described.

## Introduction

Clinical and translational research aims to move scientific discoveries to clinical innovations that diagnose, prevent, or treat disease. The Clinical and Translational Science Award (CTSA) program, funded by the National Center for Advancing Translational Sciences (NCATS), supports research training and mentored career awards at CTSA program institutions (hubs). By supporting predoctoral students and postdoctoral fellows through TL1 Awards and early stage investigators through KL2 Awards, NCATS endeavors to increase the number of well-trained clinical and translational scientists who will lead this research to improve human health [1].

CTSA hubs provide settings for timely responses to local, regional, and national health crises, such as the opioid crisis [2] and the COVID-19 pandemic. However, it is also important to understand the effects of such crises on the hubs themselves, exemplified in the extreme by the COVID-19 pandemic. Because TL1 and KL2 training environments include basic, translational, and clinical researchers, trainees and their mentors have a wide range of collegial interactions across disparate settings. Though mentored research was affected by the imposition of physical distancing and stay-at-home orders in response to the pandemic, the impact on other aspects of training and career development is unclear. In order to gain insights on how better to support TL1 trainees and KL2 scholars, CTSA Workforce Development leaders sought to evaluate the immediate impact of the pandemic on training and career development activities, maintain resilience in the research community, and meet future challenges.

## Materials and Methods

### Study Design

A voluntary and anonymous survey was distributed to the TL1 and KL2 communities via the program directors, with two reminder email messages, between April 3 and April 13, 2020. Study data were collected and managed using REDCap electronic data capture tools hosted at the University of Rochester Center for Leading Innovation and Collaboration [3]. To minimize survey burden during a stressful time and to ensure anonymity, respondents were identified only by institution and role and were asked to respond to nine questions (see below). The survey was reviewed by the University of Rochester Institutional Review Board and determined to be exempt from the requirements of the Code of Federal Regulations.

### Data Analysis

Five questions yielded quantitative data: “How are these factors negatively impacting your research?” (5-point Likert scale from 1 = no impact to 5 = total impact); “What factors have positively impacted your research?” (5-point impact scale); “As a result of COVID-19, has your institution implemented official restrictions that impact training and career development activities?” (yes/no); “As a result of COVID-19, have you discontinued any training and career development activities beyond official institutional restrictions?” (yes/no); and “Have you experienced new barriers to research?” (yes/no). Quantitative data were analyzed for the entire sample. The chi-square test was used to compare observed and expected frequencies of responses between training cohorts [4,5]. Due to the exploratory and descriptive nature of this analysis, no correction was made for multiple comparisons.

For timely qualitative data analysis, the responses to only one of four open-ended questions, “What strategies are you implementing to maintain your productivity?”, were analyzed for the entire sample and included in this report. Personal and institutional identifiers were redacted prior to analysis. After establishing a general framework for data analysis (open coding of text related to initial domains of interest), an axial coding strategy based on the grounded theory approach [6] led to specific categories, following procedures that we have used previously [2,7–9]. Two experienced coders independently assigned initial themes to each text response. Inter-rater reliability was assessed using the simple proportion agreement method rather than a more complex statistic because of the relatively large number of codes, the possibility for multiple codings within text units, and the exploratory nature of this study [10].

Preliminary qualitative analysis was performed by a single coder on a partial sample of responses to the three other open-ended questions: “What tasks have you accomplished despite the stress of COVID-19?”, “What tasks have you not been able to accomplish because of the stress of COVID-19?”, and “Please provide any additional feedback.”

## Results

### Survey Respondents

Survey responses were obtained from 58 CTSA hubs, representing 88% of the 66 CTSA hubs to which the survey was sent. Respondents included 238 TL1 trainees and 229 KL2 scholars. Using fiscal year 2019 NCATS appointment data to estimate the total numbers of TL1 trainees [11] and KL2 scholars [12], the overall response rates were approximately 41% (238/580) for TL1 trainees and 68% (229/339) for KL2 scholars. Text responses for the open-ended questions were submitted by 197 TL1 trainees (34% response rate) and 202 KL2 scholars (60% response rate).

**Fig. 1.**
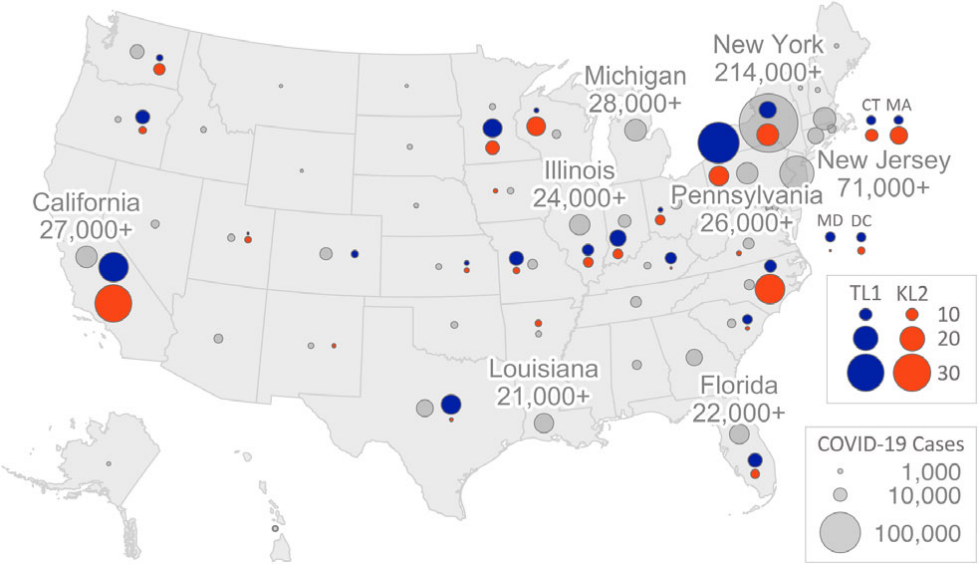
Graphic representation of the relative numbers by state of TL1 trainee and KL2 scholar survey respondents (April 3–13, 2020) and the number of COVID-19 cases (based on Johns Hopkins University data as of April 16, 2020, adapted from reference [13]). Blue circles, TL1 trainees; orange circles, KL2 scholars; gray circles, COVID-19 cases.

The geographic distribution of TL1 trainees and KL2 scholars is shown in Fig. 1 in relation to the approximate number of COVID-19 cases at the time of the survey [13,14]. The survey respondents represented 58 CTSA hubs in 27 states, including 5 of the 8 most impacted states at the time of the survey (California, Florida, Illinois, New York, and Pennsylvania). However, it should be noted that by the time of the survey, all non-COVID-19-related research at CTSA hubs had been suspended due to stay-at-home orders.

### Negative Impact of Pandemic on Research – TL1 Trainees

**Fig. 2.**
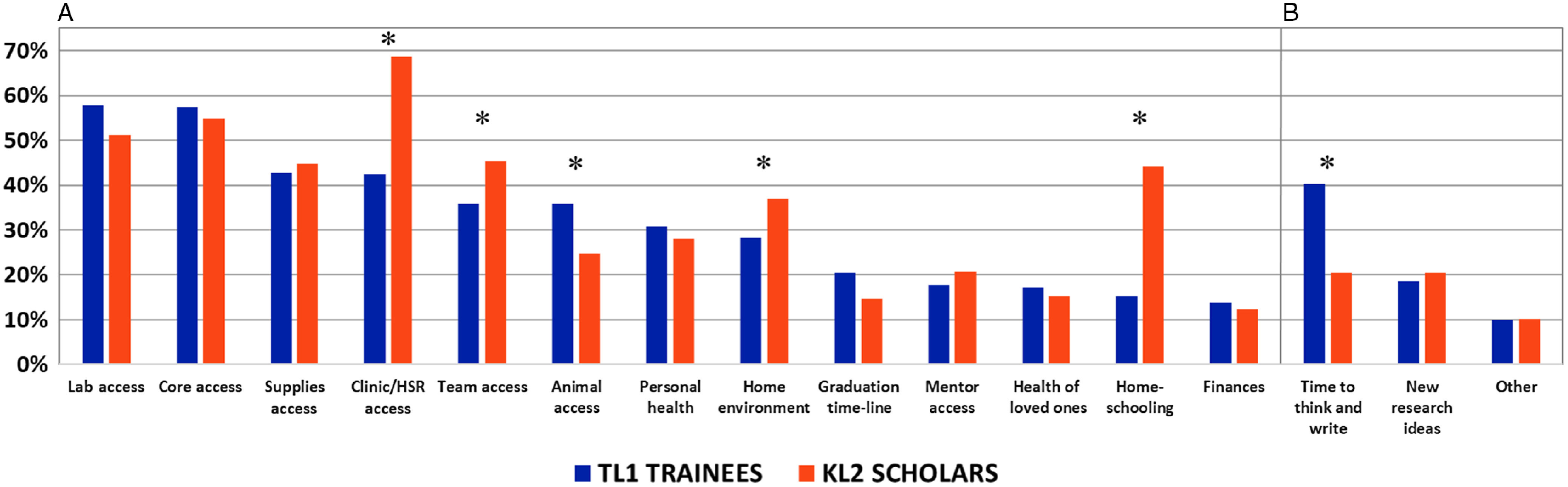
Percentage of respondents indicating high or total impact (4 and 5 on a 5-point Likert scale from 0 = no impact to 5 = total impact). (A) Responses to the question “How are these factors negatively impacting your research?”: personal/mental health, health of loved ones, finances, homeschooling, home environment, access to core facilities, access to laboratory, access to clinic/human research subjects, access to experimental animals, access to supplies, access to team members, access to mentors, graduation timeline. (B) Reponses to the question “What factors have positively impacted your research?”: time to think and write, new research ideas arising from pandemic, and other. Asterisks indicate statistically significant differences between TL1 trainees and KL2 scholars (all *P* values < 0.05). See Supplemental Tables 1 and 2 for raw data and bivariate statistical analyses.

Results from the question “How are these factors negatively impacting your research?” are summarized in Fig. 2A. The factors having the *most* negative impact and affecting more than one-third of TL1 respondents included (in descending order of response frequency): access to laboratory (58%); access to core facilities (57%); access to supplies (43%); access to clinic/human subject research (42%); access to team members (36%); and access to experimental animals (36%). TL1 trainees were impacted significantly more than KL2 scholars by access to experimental animals (*P* = 0.011). Conversely, factors reported *least* often for the highest negative impact by TL1 trainees included homeschooling (15%) and finances (14%).

Preliminary analysis of responses by TL1 trainees to open-ended questions about impact was consistent with the above quantitative results (Fig. 2A and data not shown). Impacts due to institutional restrictions reported most often by TL1 trainees included no or limited access to laboratories and experimental animals, reduced numbers of seminars and workshops, lack of clinical exposure, and less engagement with mentors and the community. Travel restrictions and conference cancellations reduced opportunities to network. Formal and informal interaction with mentors and peers was substantially reduced, and TL1 trainees had difficulty in focusing on research-related activities in their home environments. Senior TL1 postdoctoral fellows expressed anxiety over the research job market they would soon enter, and some recommended that programs immediately offer workshops and other training opportunities on hard skills (e.g., SQL, SAS) to enhance their marketability in this uncertain time. They also asked whether National Institutes of Health (NIH) funds intended for research-related expenses and travel would carry over into the next budget year of their training awards, and whether training periods and support would be extended to enable project completion.

### Negative Impact of Pandemic on Research – KL2 Scholars

The factors similarly having the *most* negative impact for KL2 respondents (Fig. 2A) included: access to clinic/human subjects research (69%); access to core facilities (55%); access to laboratory (51%); access to team members (45%); access to supplies (45%); homeschooling (44%); and home environment (37%). KL2 scholars were impacted significantly more than TL1 trainees by access to clinic/human subject research (*P* < 0.001), access to team members (*P* = 0.037), home environment (*P* = 0.045), and homeschooling (*P* < 0.001). Factors reported *least* often for the highest negative impact by KL2 scholars included health of loved ones (15%), graduation timeline (15%), and finances (12%).

Preliminary analysis of responses by KL2 scholars to open-ended questions about impact was consistent with the above quantitative results (Fig. 2A and data not shown). KL2 scholars experienced adverse effects similar to those reported by TL1 trainees, though they reported being affected more by a lack of access to clinic and human subjects research than by a lack of access to experimental animals; this perhaps reflects a difference in research portfolios, with more human subjects research among KL2 scholars and more preclinical translational research in the TL1 group. KL2 scholars more commonly worked within or led research teams, and their interactions with research technicians, research coordinators, postdoctoral trainees, and students were substantially reduced. Some KL2 scholars from clinical departments spent more time on patient care, providing either frontline care or backfilling for others. Specific challenges associated with such patient care included: increased clinical time (voluntary or mandatory) for the scholar, their mentor(s), and/or child-care partner; decreased availability of clinician mentors/collaborators; and time spent learning telemedicine technology. The combination of clinical work precedence, childcare and/or homeschooling demands, and world uncertainty were reported to increase stress and hindered KL2 scholars’ efficiency and focus. Most felt that they had less time to think and brainstorm new ideas or pivot their research to new pandemic-related studies, despite the suspension of their prior research and career development efforts. KL2 scholars expressed more concern that decreased productivity might slow or block career progression. These issues were noted especially by respondents identified by their comments as women scientists.

### Positive Impact of Pandemic on Research

Results from the question “What factors have positively impacted your research?” are summarized in Fig. 2B. Significantly, more TL1 than KL2 respondents reported a positive impact of the COVID-19 pandemic on their research by having more time to think and write (40% vs. 21%, *P* <0.001). A minority of respondents (~20%) reported developing new research ideas during the pandemic or other positive impacts (~10%), for example, writing, data analysis, and virtual learning.

### Institutional Restrictions

Overall, 87% of all respondents answered “yes” to the question “As a result of COVID-19, has your institution implemented official restrictions that impact training and career development activities?”, including 84% of TL1 trainees and 87% of KL2 scholars. Overall, 25% of respondents answered “yes” to the question “As a result of COVID-19, have you discontinued any training and career development activities beyond official institutional restrictions?”, including 23% of TL1 trainees and 29% of KL2 scholars. Overall, 79% of respondents answered “yes” to the question “Have you experienced new barriers to research?”, including 73% of TL1 trainees and 85% of KL2 scholars.

### Strategies to Maintain Productivity

**Fig. 3.**
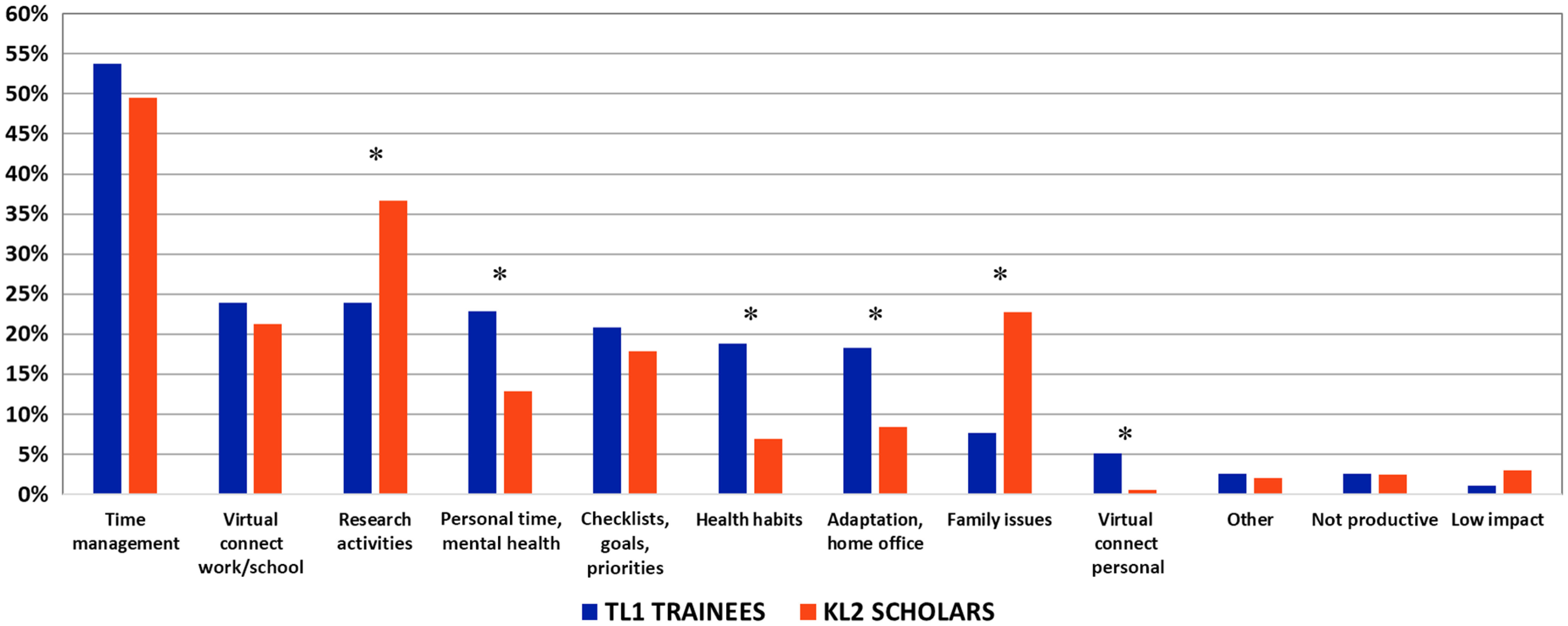
Percentage of respondents including each theme in response to the question “What strategies are you implementing to maintain your productivity?” Asterisks indicate statistically significant differences between TL1 trainees and KL2 scholars (*P* < 0.01). See Supplemental Table 3 for qualitative data, including theme definitions, frequencies, and bivariate statistical analyses.

Results from the qualitative analysis responses to the question “What strategies are you implementing to maintain your productivity?” are summarized in Fig. 3. The average concordance between raters for the TL1 and KL2 datasets was 80% (range 76–83%). The three most commonly cited strategies were “time management” (e.g., regular work schedule, routine, and self-isolation from distractions), “virtual connect work/school” (e.g., virtual connections with colleagues, check-ins with mentors/trainees, and clinical telemedicine), and “research activities” [e.g., writing papers, grant proposals, data analysis, computation, online education, new collaborations, administrative duties, balancing clinical duties, and new research direction (COVID-related or other)].

The overall frequency of the identified strategies was similar for TL1 trainees and KL2 scholars, though there were several statistically significant exceptions (*P* < 0.01). KL2 scholars identified “research activities” and “family issues” (e.g., managing homeschooling and childcare issues, hiring a full-time nanny, and scheduling around children or partner) more often than TL1 trainees. TL1 trainees identified “personal time/mental health” [e.g., taking time for yourself (hobbies, breaks, and nature walks), mindfulness, gratitude, dealing with stress, patience, and self-forgiveness], “health habits” (e.g., exercise, walking, attention to diet, sleep, and avoiding alcohol), “adaptation/home office” (e.g., adapting to home office space, varying work environment, virtual office, and allowing flexibility), and “virtual connect personal” (e.g., family, friends, social, and personal) more often than did KL2 scholars (Fig. 3).

## Discussion

Within 1 month after the World Health Organization labeled COVID-19 a pandemic, multiple impacts were felt by TL1 trainees and KL2 scholars. Educational activities shifted to being almost completely virtual, imposing a learning curve for optimizing this approach on both presenters and participants. Despite restrictions placed by institutions on research activities, it is significant to note the adaptability of trainees and scholars. The adaptive strategies described by survey respondents to advance their research suggest a number of approaches that may be taken by mentors and institutions in the event of another pandemic. Specific recommendations focus on virtual training opportunities, time management skills, mentoring, self-care, and flexibilities for training appointments.

Due to stay-at-home orders, TL1 trainees and KL2 scholars focused their research on activities that do not require on-site presence, such as writing papers and grants, data analysis, and virtual education opportunities. Training programs should be prepared to provide virtual training opportunities to advance their careers, including research skills (e.g., statistics, data management) and professional skills (e.g., communication skills, research management). The strategies cited most often by both TL1 trainees and KL2 scholars to maintain research productivity, but only by about half of them, were related to time management. Training for more effective time management could be easily implemented by CTSA programs with the assistance of institutional human resource departments, and maintaining good practices can be monitored and encouraged by mentors.

Access to mentors was not reported to be a common problem (Fig. 2), but comments to open-ended questions suggested that it was more variable, and the impact on frequency and quality of interaction is uncertain. Helpful strategies for mentors to implement include virtual lab check-ins, virtual group meetings, and frequent scheduled calls with trainees. The importance of virtual mentorship, including peer-mentorship and social connections, should be emphasized. The need for mentoring is perhaps most acute for trainees approaching times of job change who need support in planning for job searches and, in some cases, additional skill acquisition or training. There is opportunity to implement initiatives that foster collaborations among CTSA hubs to increase networking and mentoring.

Strategies mentioned more often by TL1 trainees than KL2 scholars included attention to mental health such as taking breaks, healthy habits such as exercise and nutrition, and virtual connections to family and friends (Fig. 3). KL2 scholars also described adjusting research expectations under these new constraints and better coordinating of family and childcare responsibilities. However, the fact that these strategies were mentioned by only a small minority of trainees and scholars suggests a need for more awareness about the importance of promoting self-care, which can be accomplished by a combination of institutional programs and encouragement by program directors and mentors.

Whereas many effects of the pandemic were similar between trainees and scholars, a distinct difference emerged for KL2 scholars. Homeschooling and family issues were described by three times as many KL2 scholars as TL1 trainees. This is consistent with KL2 scholars being early stage investigators who are further along in their life journey, with differing non-academic relationships and household responsibilities. However, as early stage investigators, KL2 scholars may also be more driven to maintain research productivity, as research activities were cited the second-most often for maintaining research productivity (Fig. 3).

The coordination of work-related activities with family priorities is a particularly challenging issue for KL2 scholars, complicated further for those with patient care responsibilities. As early stage investigators with time-limited KL2 appointments, they expressed concern about the possible adverse impact of decreased productivity on their long-term research goals and perceptions of funding agencies. This anxiety can be alleviated through (1) helping them use their time as productively as possible, on still-available career development activities, on their own research, or on COVID-19-related activities that otherwise align with their career goals; (2) increasing interactions and support from mentors and program directors; (3) extending KL2 appointments and research support using federal or institutional funds when allowable and possible; and (4) facilitating implementation of institutional, private, and federal grant review policies that account for this rare challenge. It should be noted that the NIH has expressed deep concern for the health and safety of people involved in NIH research and the effects of the COVID-19 pandemic on the biomedical research enterprise. In response, the NIH has introduced some flexibilities for the administration of research, training, fellowship, and career development awards [15], and provided training and wellness resources for trainees, faculty, and leaders outside the NIH [16].

Whereas this survey defined some immediate impacts of the COVID-19 pandemic on TL1 and KL2 training and career development activities, it has several limitations. Although developed by stakeholders and survey experts and pre-tested with iterative improvements before launch, this was the first use of this survey instrument; therefore, reliability and validity have not been established. Given the geographic variability in infection rates and institutional responses early in the COVID-19 pandemic, trainees and scholars likely experienced more or less severe impacts depending on their location. Aggregating results for all trainees and scholars prevented detection of pandemic severity-dependent components, and the voluntary nature of the survey allowed potential selection bias; thus, the responses might not be representative of the entire TL1 and KL2 community. The survey did not request gender or other demographic information; thus, gender-specific impacts of the pandemic could not be assessed. The absence of individual-level information, such as training stage, research focus, household composition, and patient care responsibilities, limited granular, explanatory analyses. Although it is likely that the impact on CTSA-sponsored trainees and junior investigators will extend to others at equivalent career stages, CTSA-funded trainees may not be representative of other trainees or junior faculty researchers conducting biomedical research through other funding mechanisms.

Future research will be needed to assess how these findings change over time and to capture variations in local institutional and geographic policies influencing the resumption of research and training activities. A subsequent survey after the pandemic will be modified and improved based upon the lessons from this initial survey. For example, themes from qualitative coding could become response choices for new quantitative items, items with little variance could be dropped, new items could be added to address additional domains, and information about trainee characteristics (e.g., gender, family status, and involvement in patient care) can allow more detailed analysis. It may be useful to include non-CTSA-funded researchers at similar career stages to assess whether the CTSA programs are protecting the funded trainees against adverse effects from this severe challenge.

The rapid institutional responses to the COVID-19 pandemic forced dramatic interruptions in mentored research training. Overall, at this early stage of the pandemic TL1 trainees and KL2 scholars appeared to exhibit good adaptability to the pandemic, but the long-term impact on career development remains unclear. Training leaders at CTSA hubs must be better prepared to communicate with trainees to help them adapt so as to minimize the impact of this crisis on their career development. CTSA training programs must be prepared to implement remote training opportunities and communicate more effectively with TL1 trainees and KL2 scholars about how institutions are adjusting academic programs and about funding decisions that impact training progress and career progression.

## Supplementary material

For supplementary material accompanying this paper visit https://doi.org/10.1017/cts.2020.504. click here to view supplementary material
