# Supplementary material for: Immediate impact of the COVID-19 pandemic on CTSA TL1 and KL2 training and career development
Source: J Clin Transl Sci. 2020 Jun 29;4(6):556–61. doi: 10.1017/cts.2020.504 (PMC7605410; doi:10.1017/cts.2020.504)
Supplement: Supplementary file 1 [file S205986612000504Xsup001.docx]

Supplemental Table 1. Negative Impact of the COVID-19 Pandemic on Research by TL1 Trainees and KL2 Scholars.

| **How are these factors negatively impacting your research? (1 = No Impact, 5 = Total Impact)** | | **N** | **COUNTS** | | | | | **High-Total Impact (4 & 5)** | | | |
| --- | --- | --- | --- | --- | --- | --- | --- | --- | --- | --- | --- |
|  |  |  | **No-Mid Impact** | | | **High-Total Impact** | |  |  |  |  |
|  |  |  | **1** | **2** | **3** | **4** | **5** | **N** | **%** | **χ^2^** | **p** |
| Personal/Mental Health | TL1 | 237 | 29 | 62 | 73 | 57 | 16 | 73 | 31% | 0.42 | ns |
|  | KL2 | 228 | 33 | 63 | 68 | 45 | 19 | 64 | 28% |  |  |
| Health of Loved Ones | TL1 | 238 | 78 | 75 | 44 | 33 | 8 | 41 | 17% | 0.32 | ns |
|  | KL2 | 229 | 76 | 81 | 37 | 25 | 10 | 35 | 15% |  |  |
| Finances | TL1 | 237 | 109 | 57 | 38 | 22 | 11 | 33 | 14% | 0.29 | ns |
|  | KL2 | 229 | 109 | 59 | 33 | 24 | 4 | 28 | 12% |  |  |
| Homeschooling | TL1 | 235 | 146 | 34 | 19 | 10 | 26 | 36 | 15% | 46.19 | <.001 |
|  | KL2 | 229 | 89 | 15 | 24 | 38 | 63 | 101 | 44% |  |  |
| Home Environment | TL1 | 237 | 59 | 65 | 46 | 41 | 26 | 67 | 28% | 4.03 | .045 |
|  | KL2 | 227 | 33 | 60 | 50 | 45 | 39 | 84 | 37% |  |  |
| Access to Core Facilities | TL1 | 237 | 37 | 34 | 30 | 32 | 104 | 136 | 57% | 0.31 | ns |
|  | KL2 | 228 | 51 | 20 | 32 | 36 | 89 | 125 | 55% |  |  |
| Access to Laboratory | TL1 | 235 | 65 | 22 | 12 | 26 | 110 | 136 | 58% | 2.14 | ns |
|  | KL2 | 227 | 69 | 19 | 23 | 22 | 94 | 116 | 51% |  |  |
| Access to Clinic/Human Subject Research | TL1 | 233 | 99 | 15 | 20 | 20 | 79 | 99 | 42% | 31.87 | <.001 |
|  | KL2 | 224 | 41 | 5 | 24 | 27 | 127 | 154 | 69% |  |  |
| Access to Experimental Animals | TL1 | 232 | 139 | 3 | 7 | 16 | 67 | 83 | 36% | 6.49 | .011 |
|  | KL2 | 222 | 150 | 8 | 9 | 5 | 50 | 55 | 25% |  |  |
| Access to Supplies | TL1 | 236 | 63 | 35 | 37 | 36 | 65 | 101 | 43% | 0.18 | ns |
|  | KL2 | 228 | 60 | 33 | 33 | 43 | 59 | 102 | 45% |  |  |
| Access to Team Members | TL1 | 234 | 67 | 42 | 41 | 34 | 50 | 84 | 36% | 4.35 | .037 |
|  | KL2 | 229 | 51 | 35 | 39 | 40 | 64 | 104 | 45% |  |  |
| Access to Mentors | TL1 | 237 | 58 | 59 | 78 | 30 | 12 | 42 | 18% | 0.63 | ns |
|  | KL2 | 228 | 67 | 62 | 52 | 32 | 15 | 47 | 21% |  |  |
| Graduation Timeline | TL1 | 234 | 102 | 40 | 44 | 22 | 26 | 48 | 21% | 2.64 | ns |
|  | KL2 | 218 | 155 | 17 | 14 | 16 | 16 | 32 | 15% |  |  |

χ^2^ analysis based on TL1 *vs.* KL2 counts for high-total impact (ratings 4-5) *vs.* low-mid impact (ratings 1-3).

N, number of responses; ns, not significant (p>.05).

Supplemental Table 2. Positive Impact of the COVID-19 Pandemic on Research by TL1 Trainees and KL2 Scholars.

| **What factors have positively impacted your research? (1 = No Impact, 5 = Total Impact)** | | **N** | **COUNTS** | | | | |  | | | |
| --- | --- | --- | --- | --- | --- | --- | --- | --- | --- | --- | --- |
|  |  |  | **No-Mid Impact** | | | **High-Total Impact** | | **High-Total Impact (4 & 5)** | | | |
|  |  |  | **1** | **2** | **3** | **4** | **5** | **N** | **%** | **χ^2^** | **p** |
| Time to Think and Write | TL1 | 233 | 46 | 39 | 54 | 60 | 34 | 94 | 40% | 21.39 | <0.001 |
|  | KL2 | 229 | 82 | 43 | 57 | 31 | 16 | 47 | 21% |  |  |
| New Research Ideas Arising from Pandemic | TL1 | 233 | 92 | 53 | 45 | 23 | 20 | 43 | 18% | 0.31 | ns |
|  | KL2 | 229 | 83 | 53 | 46 | 34 | 13 | 47 | 21% |  |  |
| Other | TL1 | 139 | 105 | 4 | 16 | 7 | 7 | 14 | 10% | <0.01 | ns |
|  | KL2 | 118 | 89 | 8 | 9 | 4 | 8 | 12 | 10% |  |  |

χ^2^ analysis based on TL1 *vs.* KL2 counts for high-total impact (ratings 4-5) *vs.* low-mid impact (ratings 1-3).

N, number of responses; ns, not significant (p>.05).

Supplemental Table 3. Strategies being implemented to maintain research productivity during the COVID-19 pandemic.

| **What strategies are you implementing to maintain your productivity?** | **COUNTS** | |  | **% OF RESPONDERS** | |
| --- | --- | --- | --- | --- | --- |
|  | **TL1 Trainees** | **KL2 Scholars** |  | **TL1 Trainees** | **KL2 Scholars** |
| Number of Responders | 197 | 202 |  |  |  |
| **Themes** |  |  |  |  |  |
| 1. Health Habits | 37 | 14 |  | 18.8% | 6.9% |
| Exercise, walking, attention to diet, sleep, avoiding alcohol | | | | | |
| 2. Checklists, Goals, Priorities | 41 | 36 |  | 20.8% | 17.8% |
| Checklists, to do lists, deadlines, goal-setting, prioritizing | | | | | |
| 3. Time Management | 106 | 100 |  | 53.8% | 49.5% |
| Work schedule, routine, time management, self-isolation from distractions | | | | | |
| 4. Virtual Connect Work/School | 47 | 43 |  | 23.9% | 21.3% |
| Virtually connect with colleagues, check-ins with mentors/trainees, clinical telemedicine | | | | | |
| 5. Adaptation, Home Office | 36 | 17 |  | 18.3% | 8.4% |
| Adapting to home office space, vary work environment, virtual office, allowing flexibility | | | | | |
| 6. Personal Time, Mental Health | 45 | 26 |  | 22.8% | 12.9% |
| Taking time for yourself (hobbies, breaks, walks), mindfulness, gratitude, dealing with stress, patience, self-forgiveness | | | | | |
| 7. Virtual Connect Personal | 10 | 1 |  | 5.1% | 0.5% |
| Virtually connect with family, friends, social, personal | | | | | |
| 8. Other | 5 | 4 |  | 2.5% | 2.0% |
| Sharing resources about maintaining productivity, getting help, peer/mentor accountability, asking for feedback | | | | | |
| 9. Research Activities | 47 | 74 |  | 23.9% | 36.6% |
| Writing papers, grant proposals, data analysis, computation, online education, new collaboration, clinical duties, new   research direction (COVID-related or other) | | | | | |
| 10. Low Impact | 2 | 6 |  | 1.0% | 3.0% |
| Continued research with little change, productivity is unchanged or increased | | | | | |
| 11. Family Issues | 15 | 46 |  | 7.6% | 22.8% |
| Managing homeschooling and childcare issues, hiring a full-time nanny, scheduling around children or partner | | | | | |
| 12. Not Productive | 5 | 5 |  | 2.5% | 2.5% |
| Little time, less productivity, this is a joke | | | | | |
| Total Counts: | 396 | 372 |  |  |  |
